# Degradation-mediated protein quality control at the inner nuclear membrane

**DOI:** 10.1080/19491034.2016.1139273

**Published:** 2016-01-13

**Authors:** Mirta Boban, Roland Foisner

**Affiliations:** a Croatian Institute for Brain Research, School of Medicine, University of Zagreb, Zagreb, Croatia; b Max F. Perutz Laboratories (MFPL), Department of Medical Biochemistry, Medical University of Vienna, Vienna Biocenter (VBC), Vienna, Austria

## Abstract

An intricate machinery protects cells from the accumulation of misfolded, non-functional proteins and protein aggregates. Protein quality control pathways have been best described in the cytoplasm and the endoplasmic reticulum, however, recent findings indicate that the nucleus is also an important compartment for protein quality control. Several nuclear ubiquitinylation pathways target soluble and membrane proteins in the nucleus and mediate their degradation through nuclear proteasomes. In addition, emerging data suggest that nuclear envelope components are also degraded by autophagy, although the mechanisms by which cytoplasmic autophagy machineries get access to nuclear targets remain unclear. In this minireview we summarize the nuclear ubiquitin-proteasome pathways in yeast, focusing on pathways involved in the protein degradation at the inner nuclear membrane. In addition, we discuss potential mechanisms how nuclear targets at the nuclear envelope may be delivered to the cytoplasmic autophagy pathways in yeast and mammals.

## Introduction

Misfolded and damaged proteins can be harmful for the cell. To eliminate these proteins and to maintain protein homeostasis, cells have developed an intricate protein quality control (PQC) system by which they assess the quality of proteins and take proper measures to either repair or eliminate the damaged components.^1^ Proteins can be targeted for proteasomal degradation by poly-ubiquitinylation, which is mediated by ubiquitin-activating enzyme (E1), ubiquitin conjugating enzyme (E2) and ubiquitin protein ligase (E3), the latter defining substrate specificity.^2^ E3 ligases recognize targets either directly or with the help of chaperones.^3^ While the proteasomes target predominantly soluble misfolded proteins, large insoluble protein aggregates are primarily degraded by autophagy.^4^ In macroautophagy, the cargo is sequestered within double membrane vesicles called autophagosomes, which fuse with the lysosome.^5^ Autophagosome formation requires yeast ubiquitin-like protein Atg8 or its mammalian homologues of the LC3 and GABARAP families, which become lipidated with phosphatidyl ethanolamine (PE) through ubiquitinylation-like reactions involving E1-like enzyme Atg7 and E2-like protein Atg3.^6^ In microautophagy, cargo is sequestered by invaginations of the lysosomal membrane, which then pinches off as small vesicles into the lysosome lumen^7^.

**Figure 1.**
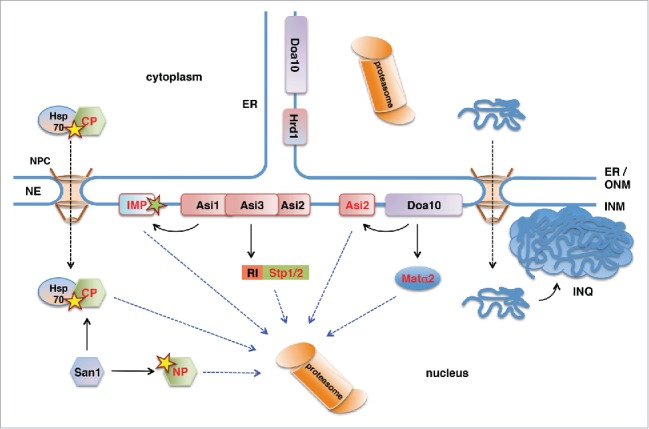
Ubiquitin-proteasome-dependent protein degradation pathways in the yeast nucleus. San1 is a nuclear E3 protein ubiquitin ligase that ubiquitinylates misfolded nuclear (NP) and cytoplasmic (CP) proteins. Delivery of CPs to nuclear San1 is assisted by Hsp70 chaperone Ssa1. Unlike the ER-membrane localized E3 ligase Hrd1, E3 ligase Doa10 localizes to both ER and the INM and targets INM protein Asi2 and transcriptional repressor Matα2 for proteasomal degradation. Asi1-Asi3 is an E3 ligase complex enriched in the INM that, together with Asi2, ubiquitinylates latent forms of transcription factors Stp1 and Stp2 via their RI degron. Asi1-Asi3 also ubiquitinylates misfolded or mislocalized integral membrane proteins (IMP) in the INM. The nucleus is also the compartment for the proteotoxic stress-induced deposit of misfolded cytoplasmic proteins and protein aggregates in the intranuclear quality control compartment (INQ).

Degradation-mediated mechanisms in protein homeostasis have been best described in the cytoplasm and the endoplasmic reticulum (ER),^8^ but a number of recent studies identified PQC pathways also in the nucleus (Fig. 1).^9^ While proteasomes are long known to localize in the cytoplasm and the nucleus,^10^ PQC pathways and their targets in the nucleus have only been identified more recently.^11^ In particular, mechanisms mediating degradation of integral membrane proteins of the inner nuclear membrane (INM) have long remained elusive. In the cytoplasm, ER-associated degradation (ERAD) is the main pathway for the degradation-mediated PQC of membrane proteins. ERAD targets misfolded proteins, but also some correctly folded wild-type proteins to the proteasome.^12^ In yeast, 2 integral membrane proteins of the ER, Hrd1 and Doa10 are the core E3 ubiquitin ligases targeting ERAD substrates.^13^ While Hrd1 primarily targets proteins with lesions in domains oriented toward the ER lumen, Doa10 targets mainly proteins with lesions in their cytoplasmic or membrane regions.^13^ Hrd1 localizes exclusively to the ER, but Doa10 was also found in the INM^14^ and targets nuclear and INM proteins.^14-16^ In addition, novel E3 ligases were recently identified that enrich at the INM,^17,18^ suggesting that several proteasomal PQC pathways exist in the nucleus and target specific sets of proteins. Furthermore, increasing evidence suggests that nuclear proteins can also be degraded by autophagy, although the mechanisms remain largely unclear.^19^ In this minireview we summarize the recently identified protein degradation pathways at the INM and we discuss potential mechanisms how nuclear envelope (NE) proteins may be targeted by autophagic pathways.

### Protein degradation in the cell nucleus

The NE consists of 2 membrane layers, the inner and the outer nuclear membrane, connected at the sites of nuclear pores.^20^ While the outer nuclear membrane (ONM) is an extension of the ER membrane, the INM has a protein composition different from that of the ONM and the ER. In metazoan cells the nuclear side of the INM is coated with a protein meshwork consisting of lamin intermediate filaments^21,22^ and lamin-interacting INM proteins and termed the nuclear lamina.^23,24^ Proteasomes are known to localize inside the nucleus in yeast and mammalian cells,^25-31^ but nuclear PQC pathways have not been described until recently (Fig. 1). A key pathway of nuclear PQC in yeast is mediated by the nuclear ubiquitin-protein ligase San1, which targets misfolded nuclear proteins for proteasomal degradation^11^ by recognizing their exposed hydrophobic regions.^32,33^ In addition, the ER integral membrane protein Doa10, which is a ubiquitin protein ligase involved in ERAD, localizes also to the INM, where it mediates degradation of transcription factor Matα2^14^ and of the INM protein Asi2.^15^ Moreover, a novel ubiquitinylation machinery that specifically localizes at the yeast INM and targets several soluble and INM proteins for degradation has recently been discovered.^17,18^ Apart from the ubiquitin-proteasome-dependent protein degradation, portions of the nucleus can also be targeted by autophagy and be degraded by the vacuole/lysosome in both yeast and mammalian cells.^34-39^ Taken together, accumulating data show that the nucleus is an important compartment for protein degradation and quality control. This is further supported by findings that the stress-induced protein aggregate deposit in yeast, which has previously been described as “juxtanuclear quality control compartment,” surprisingly localizes to the nucleus.^40^

### Asi1 and Asi3 - nuclear ubiquitin protein ligases involved in INM-associated degradation

INM proteins Asi1, Asi2 and Asi3 function as negative regulators of the amino acid induced Ssy1-Ptr3-Ssy5 (SPS) signaling pathway in yeast.^41-43^ In the absence of amino acids Asi proteins prevent promoter binding of latent transcription factors Stp1 and Stp2,^44,45^ however the molecular mechanism of this repression has not been clear so far. The first evidence that Asi proteins participate in the degradation of latent Stp1 and Stp2 in the nucleus came from findings that deletions in *ASI* genes stabilize specific forms of Stp1.^46^ Further studies showed that Asi1 and Asi3 function as ubiquitin protein ligases that ubiquitinylate latent forms of Stp1 and Stp2 in the nucleus, thereby targeting them for proteasomal degradation (Fig. 1).^18^

The role of Asi proteins is not limited to SPS-sensor signaling as additional substrates have been identified, including integral membrane proteins.^17,18^ For instance, membrane proteins Erg11 and Nsg1 involved in sterol synthesis were stabilized in *asi1Δ* and *asi3Δ* mutants,^17^ indicating that they are ubiquitinylation substrates of the Asi-complex. Protein levels of Erg11 were not affected by the levels of sterol metabolites,^17^ indicating that Asi1-mediated degradation of Erg11 is not involved in a homeostatic feedback mechanism. Rather, the purpose of Asi-mediated degradation of Erg11 is apparently to prevent accumulation of the ER-membrane protein Erg11 at the INM. The possibility that Asi-ubiquitin protein ligase has a role in clearance of mislocalized proteins at the INM is supported by the finding that vacuolar membrane proteins, which mislocalize to the ER/NE upon C-terminal epitope tagging, are also targeted by Asi1/3.^18^ In addition, Asi proteins may also recognize misfolded protein domains at the INM (Fig. 1), as the C-terminal epitope tagging of vacuolar proteins might also impair their proper folding. In support of this model a *sec61-2* mutant that becomes misfolded at high temperature and is targeted to the INM when fused to a nuclear localization signal was degraded in an Asi1-dependent manner.^17^ Taken together, in addition to ensuring latency of transcription factors in the SPS-sensor signaling, and possibly in other pathways, Asi proteins may also be involved in the removal of misfolded and mislocalized integral membrane proteins from the INM.

Apart from misfolded *sec61-2*, which appears to be a common substrate of Hrd1 and Asi1, other substrates of the ERAD ubiquitin ligases Hrd1 and Doa10 were not affected by deletion of *ASI1*.^17^ Although the Asi-ubiquitinylation machinery works with E2 enzymes Ubc6^18^ and Ubc7,^17,18^ which are also used in the ERAD pathway, Asi-mediated degradation is clearly distinct from ERAD, based on its predominant localization at the INM and its specific substrates and can thus be referred to as INM-associated degradation (INMAD).

### INM protein Asi2 – a degradation mediator and a target

Ubiquitin ligases Asi1 and Asi3 form a complex with Asi2, an integral INM protein with 2 transmembrane regions and no apparent functional domains.^17,43^ Interestingly, Asi2 was required for degradation of some substrates of Asi1/Asi3, such as transcription factors Stp1/Stp2 and integral membrane proteins Erg11 and Nsg1, but degradation of misfolded *sec61-2*^17^ and several other Asi1/Asi3 substrates^18^ did not require Asi2. Why Asi2 is necessary for degradation of some Asi-substrates, but not others is not clear, but Asi2 may recognize a specific type of degradation signal in a specific subset of Asi substrates. In the case of Stp1, the Asi-dependent degron has been defined as a 16 amino acids long sequence in the N-terminal region of Stp1, designated RI motif,^46^ but degron sequences in other substrates mediating Asi-dependent ubiquitinylation are not known.

Interestingly, we have recently shown that Asi2 itself is a degradation substrate of the ER/INM-localized ubiquitin ligase Doa10 (Fig. 1) and associated E2 enzymes Ubc6 and Ubc7.^15^ Like in most ubiquitinylated substrates, ubiquitinylation of Asi2 occurs predominantly on lysine residues.^47^ Ubiquitinylation of degradation substrates at alternative acceptor sites has been observed only in rare cases, such as in metazoan and virus-infected cells.^48-55^ Intriguingly, we found that a functional mutant of yeast Asi2 lacking all lysine residues is ubiquitinylated on atypical acceptor sites and targeted for proteasomal degradation in a Doa10-Ubc6-Ubc7 dependent manner,^47^ indicating that ubiquitinylation on alternative residues might be more prevalent than previously considered. The degradation signal that targets Asi2 for Doa10-dependent ubi-quitinylation may involve an amphipathic helix present close to the N-terminus of Asi2. Indeed, amphipathic helices are present in several other Doa10 substrates where Doa10 seems to recognize their exposed hydrophobic surfaces either directly or via chaperones.^16,56,57^ The degradation signal in Asi2 might become exposed upon changes in the molecular environment or upon loss of interaction partners. For instance, the loss of interaction between Asi2 and Asi1/Asi3 may uncover a region of Asi2 and affect Asi2 protein stability. In support of this possibility, we observed that degradation of Asi2 was faster in cells lacking Asi1 and Asi3.^15^ Our data suggest that the majority of Asi2 is ubi-quitinylation by Doa10 at the INM, although we could not experimentally exclude the possibility that Doa10 targets Asi2 also at the ER.^15^ Notably, as the inactivation of *DOA10* does not completely abolish Asi2 degradation,^15,47^ additional pathways are likely involved in Asi2 degradation, which may function in parallel with Doa10 or upon inactivation of Doa10.

### Autophagy of nuclear envelope proteins

**Figure 2.**
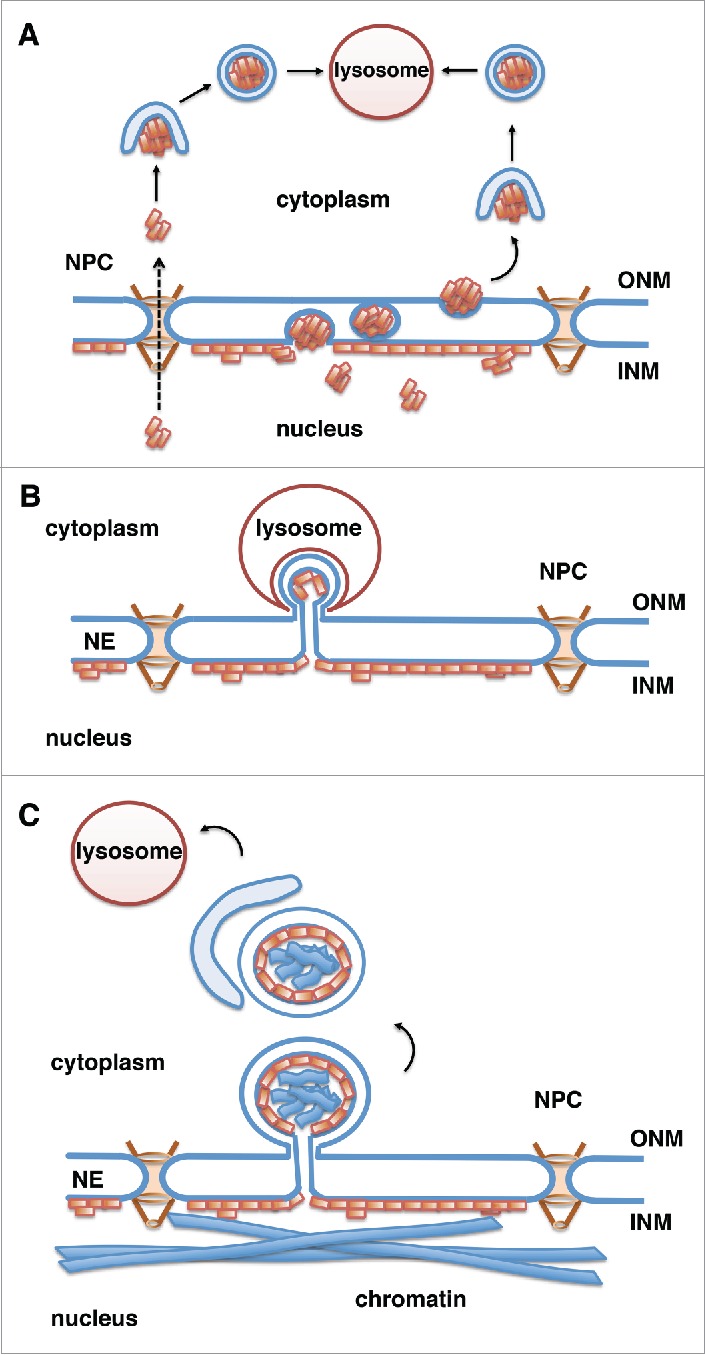
Potential mechanisms for lysosomal degradation of progerin by autophagy. Progerin (depicted by orange rectangles) is a lamin A mutant that associates with the INM. Progerin may become accessible for degradation by the cytoplasmic lysosomes in the following ways. (A) Progerin aggregates translocate to the cytoplasm through nuclear pores (NPCs) or vesicle-mediated transport through the double membrane of the NE (nuclear egress). Progerin aggregates are engulfed by the autophagosomal membrane in the cytoplasm and fuse with the lysosome.^67^ (B) Similar to piecemeal microautophagy of the nucleus in yeast, blebs of the nuclear envelope are engulfed by invaginations of the lysosomal membrane and then pinch off into the lysosomal lumen. (C) Vesicles or micronuclei bud off from the nuclear envelope, are engulfed by the growing isolation membrane producing autophagosomes, which fuse with lysosomes and deliver nuclear components to the lysosomal lumen.

In yeast, selective autophagy of nuclear material, also called nucleophagy, has been observed in a process called piecemeal microautophagy of the nucleus (PMN).^34^ In this process, which is induced by starvation, portions of the nuclear envelope form a direct physical interaction with the vacuolar membrane and form blebs that pinch off into the vacuole.^34^ A similar process that occurs after prolonged periods of nitrogen starvation was named late nucleophagy.^35^ A process similar to PMN has not been described in complex eukaryotes so far. However, an autophagy-mediated degradation of nuclear envelope proteins has recently been described in mammalian cells. Large perinuclear autophagosomes were observed in cells expressing muscular dystrophy-linked mutants of lamin A at the nuclear envelope or mutants of the INM protein emerin.^58^ Moreover, the lamin A mutant protein progerin, which is permanently farnesylated and thus tightly associated with the INM^59-61^ and causes premature aging Hutchinson-Gilford progeria syndrome (HGPS), was found to be degraded by the lysosomes in 2 recent studies (Fig. 2).^37,38^ Progerin-expressing cells exhibit abnormal nuclear shape, changes in heterochromatic marks, increased DNA damage and premature senescence,^62^ and treatment of HGPS cells with rapamycin, which is known to induce autophagy,^63,64^ ameliorated these phenotypes.^37,38^ These effects of rapamycin were linked to the increased rate of progerin degradation^37^ and reduced progerin levels in treated cells.^37,38^

The molecular details of the rapamycin-induced progerin degradation are not well understood. However, observations that inhibition of autophagosome formation by treatment of cells with 3-methyladenine or by knock-down of Atg 7^65,66^ results in impaired progerin degradation^37^ suggested that progerin is degraded by macroautophagy. Since the autophagosomes and lysosomes are present in the cytoplasm, it is unclear how progerin at the nuclear envelope is delivered to the cytoplasm (Fig. 2). Potential routes could involve nuclear export through the nuclear pore complexes (NPC) or vesicle-mediated budding of larger progerin aggregates through the NE (nuclear egress, Fig. 2-A),^67^ similar to the transport of large ribonuclear particles in Drosophila^68^ or nuclear egress of herpesvirus.^69^ Alternatively, progerin might be degraded in a process similar to piecemeal microautophagy of the nucleus, in which blebs at the nuclear envelope are surrounded by invaginations of the lysosomal membrane and pinch off into the lysosmal lumen (Fig. 2-B), although this pathway has so far been described only in yeast.^34^

Interestingly, a recent study in yeast found that in response to rapamycin treatment portions of the NE are degraded by selective autophagy via budding of double membrane vesicles from the NE and their subsequent engulfment in autophagosomes (Fig. 2-C).^36^ Degradation of specific targets in the process of selective autophagy is achieved by receptor proteins that bind to degradation targets and to Atg8/LC3 on forming autophagosomal membranes, thereby facilitating cargo sequestration into the autophagosomes.^70^ Atg39 was identified as a novel Atg8-interacting autophagy receptor at the NE in yeast.^36^ Atg39 protein levels markedly increase upon rapamycin treatment and mediate the loading of NE-derived double-membrane vesicles into the autophagosomes.^36^ Atg39-mediated nucleophagy seems to be important for yeast cell survival under prolonged nutrient-limiting conditions but it is unclear which nuclear components are targeted. In view of these recent findings on Atg39-dependent nucleophagy in yeast, the rapamycin-induced degradation of progerin in mammalian cells may occur via a similar process, including formation of micronuclei encompassing nuclear material and their subsequent engulfment by autophagosomes (Fig. 2-C). Atg39-homologues were not found in complex eukaryotes yet, however other proteins may have similar functions in mammals.

In support of a vesicle-mediated delivery of nuclear material to cytoplasmic autophagosomes in mammals, a recent study found that lamin B1, a farnesylated and INM-associated protein, is targeted by macroautophagy via blebbing of the nuclear envelope, followed by sequestration of nuclear derived vesicles into autophagosomes.^39^ In contrast to degradation of progerin and Atg39-dependent nucleophagy in yeast, which were induced by rapamycin, lamin B1 autophagy was induced in response to oncogenic insult, such as activated RAS.^39^ Oncogene-induced autophagy selectively targets lamin B1, possibly because lamin B1, unlike other lamins, directly binds to LC3.^39^ Interaction of lamin B1 with LC3 is important for lamin B1 delivery to the cytoplasmic autophagosomes,^39^ although mechanistic details of this process remain unclear.

Both autophagy of lamin B1 in mammalian cells and Atg39-dependent nucleophagy in yeast involve the generation of vesicles from the NE and their engulfment by autophagosomes. Alternatively the autophagosomal membrane could also be derived from the nuclear envelope itself as described in macrophages and other cells infected with herpes simplex virus-1.^72,73^ In these studies 4-membrane layered autophagosome-like structures were found to emerge from the NE by coiling of the INM and ONM.^72,73^ This process was named NE-derived autophagy (NEDA).^73^ Taken together, although the molecular details of progerin and lamin B1 autophagy are not completely understood, available data clearly show that nuclear envelope components might be subject to autophagic degradation also in mammalian cells.

### Concluding remarks

The nucleus is emerging as an important cellular compartment for protein degradation and PQC. Although PQC pathways in the nucleus have long been overlooked, recent discoveries revealed ubiquitin protein ligases that target PQC substrates in the nucleus and at the INM.^11,14,17,18^ Initially assumed that degradation of nuclear proteins relies entirely on the ubiquitin-proteasome system,^10^ recent findings show that nuclear material can also be subject to autophagic degradation both in yeast ^34-36^ and complex eukaryotic cells.^37-39^

Remarkably, nuclear pathways appear to be responsible for the quality control of not only nuclear, but also cytoplasmic proteins. Besides misfolded nuclear proteins, the nuclear ubiquitin protein ligase San1 also mediates degradation of misfolded cytoplasmic proteins (Fig. 1).^74-76^ Furthermore, it has recently been shown that a stress-induced protein aggregate deposit in yeast known as JUNQ (“*juxtanuclear quality control compartment*”) localizes inside the cell nucleus, and has accordingly been redefined as “*intranuclear quality control compartment*” (INQ, Fig. 1).^40^ Intriguingly, INQ serves as a deposit for both cytosolic and nuclear misfolded proteins.

Nuclear degradation-dependent PQC mechanisms are particularly important for proper function and survival of long-lived postmitotic cells, such as neurons. Unlike dividing cells, postmitotic cells cannot eliminate accumulated damage by asymmetric segregation or by dilution in cell divisions. Moreover, while in dividing cells the barrier between the nucleus and cytoplasm, the NE, breaks down during each cell division and consequently damaged proteins from the nucleus gain access to the cytoplasmic PQC pathways, postmitotic nuclei lack this possibility. Accordingly, several neurodegenerative diseases are associated with misfolding and aggregation of nuclear proteins, underscoring the importance of nuclear PQC.^77-79^

## Abbreviations

ER: endoplasmic reticulum
ERAD: ER-associated degradation
HGPS: Hutchinson Gilford Progeria syndrome
INQ: intranuclear quality control compartment
INM: inner nuclear membrane
INMAD: INM-associated degradation
JUNQ: juxtanuclear quality control compartment
NE: nuclear envelope
NPC: nuclear pore complex
ONM: outer nuclear membrane
PQC: protein quality control
PMN: piecemeal microautophagy of the nucleus
